# University students’ opinions towards mobile sensing data collection: A qualitative analysis

**DOI:** 10.3389/fdgth.2023.1125276

**Published:** 2023-04-13

**Authors:** Jack R. H. Cooper, Damian Scarf, Tamlin S. Conner

**Affiliations:** Department of Psychology, University of Otago, Dunedin, New Zealand

**Keywords:** digital health, mobile health, mobile sensing data, digital phenotyping, college, university, participation, adherence

## Abstract

mHealth researchers can now collect a wealth of data using “life tracking apps” (LTAs), which are smartphone applications that use mobile sensing to capture and summarise a multitude of data channels (e.g., location, movement, keyword use, sleep, exercise, and so on). The combined wealth of information can create digital signatures of individuals, which hold immense promise for mental health research and interventions by allowing new insights into moment-to-moment changes in behaviour and mental states. However, little is known about what a common research demographic (university students) thinks about these apps and what might factor into their decisions to participate in research using a LTA. This qualitative study ran five focus group sessions (21 students in total) to explore students’ experiences, beliefs, and opinions about LTAs to generate insights into what would make them more or less likely to participate in research involving LTAs. Transcripts were coded and examined for categories using qualitative content analysis. Important categories that emerged were privacy (although this varied based on the individual and data being collected), data security, inconvenience, intrusiveness, financial compensation, and the perceived nature of the research team responsible. On the basis of these categories, we derived seven key insights to increase student participation in research using LTAs: strengthen and communicate privacy and data security, design the app to be as convenient as possible to users, maximise passive data collection, think cautiously before tracking data perceived as “creepy” such as messages, offer suitable financial compensation, be transparent about goals and justification for data being collection to build trust, and attract participants by highlighting how the app can help them achieve their goals. With these insights, mHealth researchers can maximise their participant pool and improve this nascent and promising field.

## Introduction

Mobile technologies have become widespread and common in our daily lives. In the United States alone, smartphone ownership among adults went from 35% in 2011 to 85% in 2021 (1). This proliferation of portable technology has immense benefits for researchers including better accessibility of collecting real-time data in daily life *via* ecological momentary assessment (2, 3), use of technology for delivering real-time ecological momentary interventions (4, 5), and collection of objective, relevant data normally reserved to self-report such as sleep and exercise metrics (6, 7).

Ubiquitous smartphone use has also allowed for specialised interventions designed for smartphones, otherwise known as mHealth interventions, which can reach participants regardless of their location or current living situation, particularly relevant during the post-COVID world.

The most recent innovation to emerge from mobile technologies is the rise of smartphone apps capable of capturing multiple modalities of data. Using a variety of data formats ranging from accelerometer data to audio recordings and location tracking, these “life tracking apps,” or LTAs (sometimes also referred to as “lifeloggers”; (8), can create digital signatures or “digital phenotypes” of individuals from both more traditional metrics (e.g., exercise, sleep, location tracking) and new metrics (social media usage, time spent on device, keyword usage in messaging). Examples include StudentLife from Dartmouth (9) and the Effortless Assessment of Risk States (EARS) tool from the University of Oregon (10), with many others in development. Because LTAs can combine their varied data collection with machine learning approaches to statistical modelling, they can allow new insights into moment-to-moment changes in behaviour and mental health and provide just-in-time interventions. This is particularly useful given the ongoing reliance on digital resources caused by the COVID-19 pandemic. These capabilities are of massive interest to the health and mental health fields, both commercially and academically. While some LTAs have already highlighted their potential for research (11), they are still a relatively nascent approach.

We believe that more understanding is needed on the acceptability of LTAs, especially among university students who constitute the core research demographic. University students are a common demographic in LTA studies as they are accessed by university-based researchers. University student research pools also allow for large sample data collection, which is needed for statistical modelling of LTA data and predictive modelling (8). Additionally, students at this time of history are a unique demographic. The majority of university students are of Generation Z, an age ranging from 0 to 24 defined by growing up in an “always on” digital world and high rates of digital literacy (12, 13). It is likely they have encountered aspects of LTAs within their daily life (e.g., passive mobile sensing data collection for commercial apps such as MyFitnessPal or Apple Health) and have developed their own opinions and beliefs regarding relevant topics such as privacy concerns, data security, and usability that will influence their decisions on whether to participate. Prior research on the acceptability of LTAs is scarce and focused on *adult consumers* rather than *university student participants*, but a quick summary suggests *privacy* and *inconvenience* as major factors influencing acceptance and comfort with mobile sensing data collection (14, 15). Privacy may be less of a concern to younger demographics who have grown up in the “Age of Information,” while convenience may be particularly valued for those who have grown used to the ever-increasing support of technology for everyday tasks. Additionally, university students are often incentivised to participate in research as part of their courses (i.e., for course credit), which is a very different context to freely accessing an LTA of their own volition, as adults are more likely to do. It is important to discover the opinions and priorities of this unique intersectional demographic, so as to design LTA research in such a way to maximise participant sign up and/or volunteer buy-in. To achieve this, researchers and developers need insight into what factors influence whether university students will participate in LTA research.

Therefore, we conducted a qualitative study that aimed to (1) learn about the previous experiences university student participants have had with some elements of LTAs such as passive data collection for traditional behavioural data (activity, diet, and sleep tracking); (2) understand the opinions and beliefs participants have about regarding mobile sensing data collection and LTAs; and (3) learn what factors may make university students more or less likely to participate in research using LTAs, with focus given to privacy and inconvenience.

## Methods

### Design

This was a qualitative study involving focus groups called the “Exploring Life Tracking Mobile Apps.” Focus groups were run from December 2021 to April 2022. Ethical approval was granted by the Department of Psychology Category B Ethics with oversight by the University of Otago Human Ethics Committee (#D21/206) COREQ guidelines [specifically the 32 item checklist suggested by (16)] were referenced and adhered to where appropriate.

### Participants

We recruited 21 university students (3 male, 18 female, 0 gender diverse), with inclusion criteria being that they were (a) aged between 18 and 25 years old and (b) currently enrolled at the University of Otago, New Zealand. There were no exclusion criteria. The smaller sample size was due to challenges encountered with no-shows (participants not showing up to sessions), organisation (finding times for sessions that worked for all participants), and COVID-19 (conducting sessions over Zoom instead of in-person, which occasionally resulted in technological issues).

The University of Otago is a traditional mid-size public university (22,000 students) with a large undergraduate student population. Participants were recruited *via* a third-party website known as Student Job Search (SJS) that recruits students for paid work. The study was advertised as “Otago Student Focus Group: Exploring Life Tracking Mobile Apps.” Each participant was paid $20 for their time.

### Procedure

Five focus groups were conducted. Each group had —four to six participants and lasted 50–70 min. The researcher (JRHC) began each session by providing an overview of LTAs including a definition and some examples (see Table 1). From there, the researcher moved through a list of questions (see Table 1). The development of the interview questions was not guided by any theoretical perspective, as (1) the literature regarding LTA acceptability was scarce and did not have unifying “theories” to formulate from so to speak, and (2) the researchers wished to not artificially limit insight generation with “leading” questions (asking about *only* one expected factor such as privacy, while missing a crucial but unexpected factor like gamification). Indeed, besides the fifth question detailed in Table 1 (which were “*a priori*” questions conceived by the researchers about potential factors suggested by the literature, such as privacy and inconvenience), questions were designed to be as neutral as possible, simply asking what the participants thought about the topic. Throughout the process, the researcher (JRHC) remained reflexive regarding discussion, allowing tangential topics to emerge but steering the conversation back towards the questions asked. The researcher was flexible with what emerged during discussion, following up on and encouraging elaboration for unanticipated topics again so as to not artificially limit insight generation.

**Table 1:** List of questions and prompts in the focus group sessions.

**INSTRUCTIONS:** Today, we are discussing life tracking apps. These are applications on mobile phones/smartphones that are capable of tracking parts of your life, such as your heart rate/exercise/sleep/social media usage. Some of these apps can track this data passively, meaning it doesn’t need you to put in the information yourself, while others require your input. Finally, some of these apps aim to use your data to improve your health, whether it be by offering advice on how to improve your sleep schedule to even predicting your risk of depression from how your messaging has changed. 1. Previous experiences with LTAs the participants may have had 2. How comfortable participants felt about a particular type of information being collected (e.g., sleep vs. activity vs. location data) 3. How comfortable participants felt about a particular method of data collection (e.g., audio recordings vs. accelerometer data) 4. How likely they thought a given type of data would be at predicting depressiveness (e.g., sleep data vs. activity data) 5. How various things may factor into their decision to participate in a hypothetical experiment involving LTAs (e.g., what data was being collected, how it was being collected, financial compensation vs. course compensation, frequency of mental health surveys during study, privacy/data security settings, inconvenience or intrusiveness into daily life using the LTA)

LTAs, life tracking apps.

### Data transcription, coding, and analysis

During each focus group session, participants gave informed consent to having the session audio recorded. Prior to coding, audio recordings were transcribed verbatim using a third-party automated service known as Otter.ai, with the researcher manually adjusting any mistakes made and improving readability. Speakers were also given code names. Only the primary researcher, JRHC, was involved in coding the results. The primary goal for the study was to explore the experiences, opinions, and attitudes university students held regarding LTAs, and how this may influence their decisions to participate in LTA research. This objective is most appropriately accomplished by utilising qualitative content analysis. Qualitative content analysis is a method of analysing qualitative data by that “focuses on subject and context and emphasizes variation, e.g., similarities within and differences between parts of the text … It offers opportunities to analyse manifest and descriptive content as well as latent and interpretative content” (17). What is meant by this is that qualitative content analysis can focus on the obvious, “surface level” content of a text (the manifest content) and/or the underlying meaning known as the latent content. Manifest content and its abstraction into categories often answer “what” questions (18). Given the study’s goals, to understand what opinions/beliefs participants had and what factors may influence their participation in LTA research, focus was given to the manifest and descriptive content of the data (what was literally said), sticking closer to phenomenological descriptions and concrete analysis. This was achieved by utilising the framework suggested by Graneheim et al. (14). The framework consisted of (1) selecting the unit of analysis for the study (the transcripts of the conducted interviews), (2) creating “meaning units” or “codes,” namely, extracts of words/sentences from the transcripts that relate to each other in both content and context, (3) creating categories, namely, grouping codes that share a commonality together, and (4) creating subcategories at varying levels of abstraction when the overarching category was too general to discuss at the level of detail desired.

## Results

All participant names have been codified to preserve anonymity.

### Categories identified in the analysis

Four major categories were identified. These categories were often broad and did not encompass the nuance of the data.

Therefore, subcategories were also created.

#### Category 1: Previous experiences with mobile sensing apps

Most participants had previous experiences with apps utilising mobile sensing data, particularly exercise and diet tracking. While no participants had experiences with actual LTAs (apps capable of tracking multiple types of data such as exercise and sleep), they had experience with the concept of having their personal data tracked by apps. The main subcategories extracted from analysis were as follows:

##### Category 1a: Positive previous experiences with exercise tracking

For the apps using mobile sensing to track exercise, previous experiences were positive. Some appreciated the comprehensive data the apps collected about their exercise, and how said data was useful to their overall goals:

• *See, I wanted to upgrade to a Garmin because it can track even in more detail your exercise, like when you're running, it'll give you a stride length, which I just think is insane*—Copper (22F).
• *I used to use Strava quite a bit back when I was still running. That was, that was quite useful for me*—Iron (18M).

Others enjoyed the apps confirming that they had been active, and revealing their behavioural patterns to them:

• *Like for the Apple Watch, that tracks my exercise and fitness stuff, I like it. Because just so that I know that I've been active*—Zinc (22F).
• *Apple Health's really good for getting like your trends of what you do in a week. That's really good*—Iodine (21F).

##### Category 1b: Negative previous experiences with diet tracking

For apps designed to record diet and nutrition data, experiences were entirely negative. The only app mentioned by name was MyFitnessPal. Some female participants disliked the impact tracking their food had on their mental health. However, this may not be a gendered effect and may simply be a consequence of the largely female sample.

• *But I've tried like, my fitness pal, or something like that that track your calories, but I feel like that makes me more anxious about what I eat. So I deleted it—*Zinc (22F).
• *But (with) My Fitness Pal, I get into really unhealthy habits—*Copper (22F).

Others experienced frustration with what the app asked them to do:

• *I didn't really like my fitness pal, because it gets quite picky on listing all of your food that you ate and stuff. And it's just frustrating*—Selenium (23F).

Others found the active data collection of the diet apps was too demanding, particularly regarding caloric and nutritional information which may be difficult to estimate/track:

• *I've also used some apps that track like your diet and calories. Can’t remember what they're exactly called. But a lot of those is like manually you had to put it in. So it was quite hard remembering like exactly what you've eaten that day. Yeah, gets a bit confusing—*Gold (21F).

#### Category 2: Attitudes and beliefs about mobile sensing data collection

Participants held a wide variety of beliefs and attitudes regarding LTAs. The major subcategories that emerged were as follows:

##### Category 2a: Believing that mobile sensing data collection is inaccurate

Several participants mentioned that they believed mobile sensing data apps provided them with inaccurate information. They seemed to view this inaccuracy as inevitable/part of the process:

• *I feel like it wasn't very accurate in how many [steps] it said because there were differences between like my Fitbit, and what the app said*—Potassium (21F).
• *I think that's actually similar to the health app on like your phone. Because I can say that on like, when I checked that whenever it's different to what it says on my Apple watch. And like, I know, it's not always going to be completely accurate. But I can just tell that the health app on your phone seems to overestimate things that such as your steps, and things like that*—Diamond (20F).
• *With my Fitbit, I just I know that it is probably not accurate at all. I was in the car on a road trip and just while we were driving it said that I was doing 500 steps, which obviously I wasn’t, I was seated in the car*—Quartz (19F).
• *The app I used kinda like recorded sleep by taking the time we like turned your phone off and wasn't using your phone until we got on it in the morning, which I was more comfortable with. But also like it's not accurate because not everyone falls asleep as soon as they get off the phone. Not everyone checks your phone as soon as they wake up, although a lot of us do. A lot of people like particularly for me, it can take me like a couple of hours to fall asleep*—Topaz (20F).

##### Category 2b: Apathy or resignation regarding their personal information being collected

Interestingly, some participants expressed a form of apathy regarding the fact that mobile sensing data apps may collect personal information about them, citing that such collection occurs already and is too difficult to resist:

• *It's kind of complicated to figure out what is being shared and what isn't. And generally, I just can't be bothered, because I kind of figured that even if I did try and bother, like, there's probably other apps that are getting the information and sharing it that I don't know about—*Magnesium (22F).
• *I feel like they're kind of …. most websites for like cookies and stuff are doing that anyway. So, I don't really see much of a difference*—Silver (20F).

There was also cynicism that application actually honoured their promises to not collect information, driven in part by knowledge of mainstream controversies regarding data privacy with companies such as Facebook:

• *But the thing is, is that, like, I feel like that apps can claim that they respect privacy, like how Facebook claims that they respect privacy, and then like creepy ads come up. So, I don't know if you can fully believe them, claiming that without any evidence. It is important, but like, you still feel a fear about it, because they can make a ton of claims, but there's no proof that they're not looking into your data*—Topaz (20F).
• *And then there's no telling if those companies are also … because most of those companies are like probably free, you know, free apps that you just download. So then, and then people who are prone to like sleep talking and stuff, would probably, you could just sell that kind of data off to other companies—*Iron (18M).

##### Category 2c: Believing LTA apps were “invasive”

Many participants raised issues about having certain information tracked, and the methods used to collect it. In particular, many found their location being tracked to be a “creepy” uncomfortable prospect:

• *Yeah, I think maybe for me, it's about the movement and the GPS… the location tracking, like that? Because something kinda like, for example we all use Snapchat at some time in our life. I forgot to turn off the location. And some of my friends like, “Oh hey do you wanna … are you there are you there? …. It's so creepy, and I went “oh I turned on my location,” it's like … but yeah, but like, the app is checking itself, that's kinda creepy for me—*Clay (20M).
• *(In regard to how they felt about their location being tracked) I felt a little iffy. It's the same kinda note that it's tracking you*—Copper (22F).
• *I'm kind of same with location, like, usually, if an app asked to like, share my location, I usually say no, because I just don't know how they're storing that data. And whether that can be like hacked into somehow. Yes, I'm just not usually comfortable sharing my location, especially if I don't know the app*—Gold (21F).

Others were concerned about having sleep LTAs recording audio:

• *If it was watching me knowing when I'm sleeping and listening to that, I find that a little creepy*—Copper (22F).
• *The recording through the night, that's kind of weird. I wouldn't like them*—Magnesium (22F).

When asked what they thought about an LTA that could track one’s messaging on their mobile phone, participants were universally uncomfortable with the idea:

• *From a personal point of view, it would feel way too invasive … You just feel totally watched*—Feldspar (22F).
• *(Referring to an LTA potentially tracking messaging) I'm really not comfortable with that*—Gold (21F).
• *There are really personal topics that I discuss and probably wouldn't want someone reading*—Copper (22F).

#### Category 3: Factors affecting participation in research using LTAs

The participants were given a hypothetical scenario where they had the option to take part in research involving an LTA that was aiming to predict depression *via* the data it tracked. This is very similar in concept to the real-life app StudentLife created by The University of Dartmouth, which has seen significant participation from university students. They were then asked how different factors might influence whether they chose to participate in the hypothetical experiment. These factors included what the app might track (e.g., sleep data, location data, messaging data), who was involved in running the experiment (e.g., a large company vs. a university research laboratory), and how the LTA approached certain concepts relevant to digital literacy. This section of the procedure is the only one where the researchers employed a deductive or “concept-driven” approach, where four main factors were conceived of prior to the focus group, with participants being asked to explicitly comment on how these factors would influence their decision to participate in the hypothetical scenario. These factors (reported in the format of categories below) were as follows:

##### Category 3a: Privacy is important to most, but how important it is depends on the context

Defined to the participants as how the LTA approached protecting one's identity from the researchers/app developers when analysing their personal data), privacy was important to most participants, but how important it was varied based on the individual, the data being collected, and the presence of de-identifying measures. For some, privacy was important on principle and was explicitly stated as being highly influential on their decision to participate.

• *That (privacy) is a very big part for me because, like, behind the door, those company, I don't know what they do with my data, my messages, my pictures*—Clay (20M).
• *Yeah, I'm a bit of a I'm a bit of a privacy freak. So, I think anything that's like, that goes further than just manually inputting your information is just no-go territory for me*—Limestone (20M).
• *Oh, yeah, like, for me, it's probably like, quite significant*—Iron (18M).
• *Yeah, I think it's quite important*—Magnesium (22F).

For others, privacy was of flexible importance depending on the type of data being collected: exercise and sleep data were mentioned as being acceptable, while messaging data were explicitly mentioned as not acceptable:

• *It would matter for me a bit, depending on what type of data they were collecting, like, if it were my heart rate, I wouldn't really care if anyone knew what that was. But whereas if it was like messages or anything like that, then I'd care a lot more*—Potassium (21F).
• *I'd be mainly fine with sleep, fitness, all that. I mean, it wouldn't be the way that they recorded it. If they were like, like, recording it in a strange manner that made me feel uncomfortable, then I probably wouldn't do it. But usually that I would be fine with participating in a research study like that. However, if it was more like, phone based, like, what are you doing on your phone, texting? What apps you use what you're searching up on the internet? I feel like that's a breach of privacy. So I probably wouldn't participate in a study like that*—Quartz (19F).
• *I'm quite an open person. And so if the study is on how much I exercise, I'm quite happy to admit I don't exercise a lot. And so if that's like, around, I'm kind of I'm-I'm quite chill. But you know, there are certain things that could be researching that I want to remain very, very private. So I think the levels of privacy can differ depending on the topic of the research*—Chromium (23F).

Some participants expressed that they would be more likely to participate if appropriate de-identifying measures were in place. This was interesting as these participants were not valuing privacy on an abstract level but a concrete one; as long as there was no way to have their information linked to them, they did not mind that type of data being collected:

• *So like, were the, the participants were de identified. So I'd be fine. Whether it was like commercial or private, or research or whatever, as long as there was like a clause that said, like you will not be able to be identified from the data that's been collected*—Feldspar (22F).
• *It doesn't really worry me, because I would—It's probably quite like naive or ignorant of me. But I'd like to think I just, I'm just a number in the data. So not necessarily looking at like what [NAME REDACTED] did that day and specifically looking into me as a person. So yeah, it doesn't really worry me*—Selenium (23F).
• *I’d just like to know that there would be no identifiable factors used really*—Iodine (21F).

##### Category 3b: Data security is universally important

Defined to the participants as how the LTA approached protecting their personal data from third parties) is important, data security was unanimously important to participants. For some, it was an explicit concern over their personal information being used to contact them by advertisers:

• *Like, especially if they're using that information to sell that data on to companies to advertise stuff to us. So, if we go into a sports shop, they know that we might like sports or that or like Hallensteins or Glassons, like that. They'll cater-they'll sell that data, and then cater their advertising to us, which I feel uncomfortable about—*Limestone (20M).
• *Yeah, I think it's quite important, because it's really annoying. Like, when you get a random, like, for instance, your number getting given out or whatever, and you get random spam calls and like emails, stuff—*Magnesium (22F).

For others, concern arose from having their information “out there” being used for unknown purposes:

• *For me out of the list, you said that one will probably be the most important. Just because that would mean that you wouldn't know what would be happening to it like at all—*Silver (20F).
• *It just depends on like, what it actually was that they're collecting. Like, if it was more personal data, I would want to know that it was safe. And if it's just general stuff, then I wouldn't care so much—*Iodine (21F).
• *I think that's a really big thing for me. Even if it's something like for example, like this study where it's like, I don't… not really too fussed about it like privacy, because it's nothing super personal, like kind of information. But even with that, I kind of do a lot of things to try to prevent any sort of, like third parties from accessing my information, just because you never know what they'll be able to do with it. See, for me, like, I'd rate that very important. It's probably the top one for me—*Gold (21F).

##### Category 3c: Inconvenience (or lack of it) is possibly the most influential factor for participation

Defined to the participants as the difficulties associated with setting up and using the app, such as battery drainage, crashes, confusing login procedures, etc., inconvenience was disliked by all. Many participants explicitly mentioned that an inconvenient LTA would vastly decrease the likelihood of them participating or adhering to the study's protocols, citing that the whole point of LTAs are to be convenient:

• *The appeal of these kinds of apps is that you can turn them on and you don't have to think about them—*Iron (18M).
• *I think the only factor for me would be just how much it interrupts my life. Like, I would find it quite interesting what you're saying with the trends and stuff. But if it was something that was distracting me from my uni work, or like, making my job kind of be a second priority, then it wouldn't be worth it in the long run—*Silver (20F).
• *I think if it was an ongoing thing, I know myself well enough to know that if anything's difficult, and it takes a long time to do every time, I'm not gonna do it. You know? So that's definitely a deal breaker for me—*Feldspar (22F).
• *Yeah, I wouldn't mind just probably whatever was easiest, like, I probably wouldn't be bothered to like always go in somewhere. Like, it'd be easy if it was just like automatic like a Fitbit—*Potassium (21F).
• *I would just note on the draining of battery life, that would really annoy me. Like if I was going out for a massive day and I was taking part in the study, and I need to entering all the stuff, but it's going to drain my battery, it would probably really annoy me—*Copper (22F).
• *Easy to navigate and not drain battery life is kind of what you're looking for in, like a fitness tracking app, right? You want it to be easy to use—*Copper (22F).
• *I'd still want to carry on my normal life with it automatically tracking the data that it collects, but like, as you say, then some privacy issues come about with that—*Limestone (20M).
• *If it was ongoing, it would be annoying. If it was a short-term thing it probably wouldn't bother me so much—*Iodine (21F).
• *I probably agree in the sense that I wouldn't probably do it if it was going to be draining my battery life constantly. Because I know that I can-well, with my current routine, my phone battery gets me through the whole day, I don't have to worry about bringing chargers or any of the above. So it would be really annoying if I then had to factor that in, just to be able to do my normal everyday stuff—*Selenium (23F).
• *As soon as I download an app, I'll know straight away if I kind of like it or not, like, I'll be like, Oh, this is not working. It might be lagging. You just won't understand like, how it works with the main pages. And if like because usually I will be downloading a sort of fitness app or anything like that for a specific purpose to track something. And if I can't immediately do that, then I'll probably just like delete the app straight away—*Quartz (19F).

However, some mentioned that for research purposes they would be willing to endure a certain level of inconvenience, particularly if it was “for research” or about a topic of personal interest. This is of particular interest as it is a unique insight specifically about research contexts:

• *I think just in like day to day life. I would just get annoyed and give up on it. But I feel like I would be a bit different if it was for research. Because if I think it'll depend on what the research on and whether I'm actually interested in that topic myself. If I was quite interested in the research, I actually probably would just power through it—*Gold (21F).
• *Yeah, I feel like it's the same, like if it's just for myself, and it's more of an inconvenience. Then again, then I probably wouldn't bother. But if it's for research, I feel like I've been more willing to try a bit harder to make it work—*Pyrite (19F).

##### Category 3d: Intrusiveness is seen as helpful in moderation

Defined to the participants as the extent to which using the LTA would interrupt their daily lives, such as alerts, notifications, time spent filling out surveys, etc., intrusiveness was something of a Goldilocks factor: not too many, not too little. Too many notifications was explicitly mentioned as a potential barrier to participation, but having some was viewed as helpful towards achieving goals:

• *If there's too many notifications. I'll just turn the notification off … well, I'll just delete it—*Zinc (22F).
• *Yeah, I think there's like a fine balance. Like, it's not a bad thing, having like the odd notification or something like that. But if it's like constantly in your face, it's really annoying—*Bauxite (21F).
• *I think the more intrusive it is, the more it kind of feels like a chore. Where if you're getting like, you know, every like; even every like, second third hour, I just, I just want to delete it right away—*Iron (18M).
• *I think it also depends on the purpose of the app, like, is the app just supposed to be tracking you? Like, so say, like a health app kind of thing. Like that might ping you. Like, I know, mine pings me. And it's like, Oh, you haven't actually synced your like data. From your watch, like once-a-week kind of thing. And like, for me, that doesn't really feel like a big deal—*Feldspar (22F).
• *It would be kind of annoying, getting like lots of notifications, saying that you're depressed or something like that, when you didn't think you were—*Silver (20F).
• *When you say intrusiveness, I just think of Apple Watches and how they remind you to stand up every hour. And that really starts to annoy me some days—*Iodine (21F).
• *Apple Watch does do that with some things. I found Nike Run Club quite good. It's recognized that I haven't gone for a run through the app for a while. So it suggests a guided run for you to do but you can just decline. Yeah, but then on the other hand with my fitness pal, when I was really trying to get into tracking all my phone, It didn't remind you very often? Or it reminded me like really inconvenient times. So I never really recorded much. So I wasn't consistent with because I kept forgetting. So it's kind of hard to get the balance of enough to be usable, but not too much to be frustrating—*Selenium (23F).
• *The same goes for like, Carb Manager. That reminds you, like three times a day to log your food. And I feel like that just becomes intrusive and a wee bit annoying—*Iodine (21F).
• *I think it's important that they give you like an option. If you want the notifications and stuff. If it's really easy to turn the notifications off, that's good. And also some notifications can be useful, like some apps can give you like reminders to do certain things, as well, like taking medication or something. Yeah, that's kind of just having the option of being able to turn it off. Because if it doesn't let you that's really irritating—*Topaz (20F).

##### Category 3e: What questionnaire frequency qualified as “too much” was highly individualistic

Participants were also asked how many mental health surveys would be acceptable to them before becoming “too much.” Opinions varied across individuals, with some participants being okay with daily surveys while others barely tolerant of weekly administration:

• *I know daily would be too much for me. I'm not struggling with depression but even daily, I will just be like … (another participant: “15 questions”) … yeah, it'd be really admin. And then “ugh, I have to do this this every morning”, whereas if it was every couple of weeks, I’d be like “Ah yeah I'll fill out this questionnaire”—*Copper (22F).
• *I'd be comfortable with like, once to twice every day. Probably not more than that—*Feldspar (22F).

#### Category 4: Additional factors

Participants were also asked to offer up any factors they could think of that would influence their participation in LTA research. The factors they offered up were conceptualised as subcategories and were as follows:

##### Category 4a: Financial compensation is a guaranteed way to increase participation or adherence

Overwhelmingly, the participants mentioned financial compensation as something that would increase their participation/adherence even if they otherwise found the task difficult or effortful:

• *If I'm getting compensated for it financially, I'll do it every single day, honestly—*Iron (18M).
• *I think if your focus group is students, then probably financially, I'd say. So, money or fees off your course for that semester. I don't know how- it's hard to know what your budget is like … I reckon $5 a day—*Limestone (20M).
• *If there were a financial incentive I would be like, more than happy to do it every day—*Potassium (21F).

##### Category 4b: The perceived size and nature of the research institute involved is important to participants

For some, smaller companies or universities were preferred for perceived authenticity and personability. The local university that all the participants attended was mentioned multiple times, being perceived as “smaller” and more “personable”:

• *I feel like if it was a small little [University] group, I'd feel like I'm supporting local people doing their research—*Copper (22F).
• *I’d do it for the uni, but I wouldn't do it for a tech company—*Limestone (20M).
• *Um, I think because like if it was some big company, you know, like Google, Google Apps, or something that obviously quite like statistic based, you know, like they’re always looking at the statistics of who's using the apps, how many people, you know, just for the business? And it probably shows that it's like, it's not unethical, but it'll probably see they’re in it just for how much data they can get whatever they can get off you. You know, whereas the smaller- like the [University] thing you said, I'd be a little bit more comfortable with that. Because you're kind of only sharing to a smaller, like, group of people that aren't so- yeah I don't know if that makes sense—*Diamond (20F).
• *I feel like I'd be more likely to do it if it was like a smaller group of researchers rather than a big company. Just I don't know why—*Iodine (21F).
• *Yeah, I definitely agree with that. I'd much rather, like I'd be more inclined to go for an app that has those developed by like a smaller group, especially if it's being used for like, research purposes, rather than just a giant company—*Feldspar (22F).
• *I would be quite comfortable with like a research thing for the uni because that I know what their intentions are and why they're doing it. I would still be comfortable doing something for like Google … Like it's less personal. And they're not going to know who I am at all. But at the same time, I don't really know their full intentions. I* guess *because they are a bit more commercial. So they could have kind of other intentions that they're not really letting you know—*Gold (21F).

For others, larger companies were preferred thanks to their perceived legitimacy and capability:

• *All nowadays, we’re all using like Outlook and Gmail and stuff like that, so we start trusting them also …. So maybe we have Google or Facebook, like “Hey we have this new thing with Otago research” then we might have like that belief. And make it easier to ease into the research—*Clay (20M).
• *I feel like quite influenced by it (company size), because some of the privacy around smaller companies sometimes can be a little harder to protect, because they don't have as much like security around their data and all that—*Dolomite (21F).
• *I feel like when you are doing a much larger study, probably with a company where it's not so much like in person, you can just be one of the many. And you kind of feel like it can be less personal. Whereas at the university, like you know, you go in to do the study, you meet the people, you sit down with them, depending on what you tell them, maybe you'll see them around town later on. In that respect, like bigger, bigger studies, and you can feel like one of the MANY like they're not even going to know or remember who I am, never recognize me like nothing like that. You can somewhat feel more comfortable with that—*Chromium (23F).

##### Category 4c: Transparency about research goals was also influential to participation

Participants expressed a desire to be informed of how their information was going to be used by the researchers:

• *I'd want to be knowing what their goals were for the research, what they're aiming to achieve, and if they're not willing to share that with you, I wouldn't want to be a part of that. Because it's your information—*Copper (22F).
• *What would make me like a little bit more comfortable with like sharing that information, or like them telling me the type of experiment I was part of? Like the type of research experiment, instead of just being like, kind of blindsided and being like, are, you know, part of something that you don't actually know what they're looking for—*Diamond (20F).

##### Category 4d: Perceived relevance to daily life increases the likelihood of participation

Participants were more likely to participate if they perceived the LTA/research as relevant to their daily life:

• *I'd be more likely to participate if it was something that I could like, say for the life tracking apps. If it was an app that I could see myself using, even if it wasn't research related—*Bauxite (21F).
• *Unless, if like, for instance, I wanted to track that myself or want to, you know, like, if there was, if it was for my benefit, like for my mental health, then maybe (in regard to participating)—*Magnesium (22F).
• *I think for making me more likely to participate is if it was something I was interested in—*Gold (21F).

## Summary

Table 2 presents a summary of the insights from the focus group categories that should be considered to maximise participation in research using life tracking apps with university students.

**Table 2:** Seven insights to increase university student participation in research using life tracking apps.

1. Strengthen and communicate privacy and data security. The importance of privacy measures and data security for participation varies based on what data are being collected (e.g., low for exercise data, high for keystroke information). It also varies on an individual level: some participants were ambivalent while others were “privacy freaks.” 2. Prioritise designing/utilising convenient LTAs to minimise inconvenience, thereby maximising potential participant uptake and adherence. However, some inconvenience may be tolerated given the unique context of participating in research, and this tolerance can be increased *via* financial compensation. 3. Maximise passive data collection to minimise perceived effort involved in participation. 4. Minimise tracking certain types of data perceived as “creepy” unless vital to research question (e.g., location data, keystroke information, audio recordings). 5. Offer suitable financial compensation. Even small amounts incentivise adherence and participation among university students. 6. Be transparent regarding research goals and reasoning for the data being collected in order to overcome embedded distrust regarding privacy and data security. 7. Attract participants by tailoring study advertisements to highlight how the study can help achieve goals/behavioural insights.

LTAs, life tracking apps.

## Discussion

### Categories 1 and 2: Previous experiences and opinions/beliefs about LTAs

As expected, participants had encountered mobile sensing data collection before, particularly in the form of commercial apps. Reflecting the heterogeneity of mobile sensing data collection methods, experiences were varied. Some apps were perceived as helpful and simple to use, particularly the exercise apps that lend themselves to passive data collection *via* (relatively) accurate methods of data collection such as accelerometers. Others provoked anxiety and stress within users due to their emphasis on cumbersome active data collection, such as the nutritional tracking apps. This reflects a common-sense notion that passive data collection is better received by participants because it requires less effort from them, which may in turn improve acceptability and reduce non-adherence. Researchers should consider maximising the passive data collection components of their LTAs in any way possible, so long as the method still produces accurate/reliable results.

Relatedly, participants believed the data collected by mobile sensing methods to be inaccurate. This belief was based on their previous experience with exercise apps overestimating their activity. Despite this, participants also said they found the data collected by exercise LTAs useful in pursuing their goals and that they were excited to have the app show their activity. This may indicate that participants do not care if mobile sensing data collection is inaccurate, so long as they perceive it as helpful towards achieving goals or generating insights into their own behaviour. Researchers may consider tailoring study advertisements to include mentions of how LTAs can help with goal achievement/behavioural insights to attract participants. However, some evidence was found to suggest that LTAs may introduce iatrogenic effects or even potential contraindications within users. Specifically, participants mentioned that using LTAs with diet-tracking components actually worsened their mental health, despite the typically seen protective relationship between engaging in “healthy behaviours” such as improving one's diet and mental health. This disruption is likely due to the LTA unintentionally intensifying unhealthy mindsets and behaviours, such as perfection motivation, body image issues, obsessive tracking, restrictive eating, over-exercise, and anxiety. This disrupted protective relationship is a unique finding in LTA-based research and has been observed in a similar context of users following Instagram “health influencers” (15). Further research into why LTAs disrupt the protective relationship between healthy behaviours and mental health for some is strongly recommended.

A prominent category that emerged from these focus groups was how some data collection methods/types of data were considered “invasive.” Participants found the idea of audio recordings of them sleeping to be uncomfortable, and similarly viewed location data and keyword usage metrics as “creepy.” However, all of these methods of data collection have unique advantages in creating a holistic digital signature (for example, location data may provide invaluable insights into how often an individual leaves the house and for what purpose).

Researchers may need to weigh the benefits against a decrease in participation from their inclusion, particularly relating to keyword tracking in message content. In fact, *all* participants showed a strong dislike to the idea of their messages being tracked, citing the personal sensitivity of the content as well as just disliking it on principle. This concern maintained despite the researcher explaining that in the hypothetical scenario, all the LTA was doing was tracking whether certain keywords were said (e.g., “suicidal” and “depressed”) and tallying the count.

Researchers may have to consider that keyword tracking may be a bridge too far for most participants, and instead rely on other indirect, less invasive measures instead (such as time spent on social media or using the device).

### Categories 3 and 4: Factors influencing LTA research participation

The participants gave a wealth of information about what might make them more or less likely to participate in LTA research.

Regarding privacy, some participants showed apathy regarding their personal information being collected, citing the belief that the data are already being collected by other parties. This apathy is in sharp contrast to the cautiousness some other participants showed about having their data collected, and the lengths to which they went to avoid having it collected. This suggests that some participants may be comfortable with LTA research regardless of its approach to privacy, while others need assurances of best practice to participate. However, nearly all participants believed that companies may lie about what information they are collecting and what they will do with it, even after assuring a commitment to privacy. This cynicism may be hard to soften given the well-known examples of companies using collected personal information for unethical purposes, such as the Facebook-Cambridge Analytica scandal. Regardless, it would be prudent for LTA researchers to emphasise their commitment to privacy and data security in study advertisements to potentially convince “middle ground” students to participate. Additionally, being transparent regarding the goals of the research may endear the researchers to participants and ease their concerns; participants explicitly said this transparency may make them more likely to participate. Ultimately, this insight tells us that researchers should prioritise maintaining privacy and communicate this priority to maximise intake. This ensures that “privacy freak” students participate, as well as potentially convincing middle ground participants, without alienating the ambivalent students. This focus on privacy should be signposted in advertisements.

Data security was also important to participants, on par with privacy. This may be due to the two concepts being conceptually similar, although participants did understand the distinction when responding (e.g., having their data at risk from being stolen by advertisers as opposed to the researchers seeing their personal information). Participants expressed several negative emotions should data security be breached: fear, discomfort, and annoyance. Annoyance is a unique emotion that was not mentioned when discussing privacy; participants gave the example of having telemarketers or advertisers contacting them using stolen personal information. The threat of annoyance may be important when participants sign up for LTA research. Directly addressing this concern (e.g., assuring participants that their data will not be sold to advertisers and cannot be stolen) may help increase intake, but it is possible that the well-known controversies relating to data security (e.g., Facebook, Sony, numerous others) may render these good faith efforts moot.

Inconvenience unsurprisingly had a strong effect on whether participants would participate. Almost all participants explicitly stated that should using the LTA research be too difficult or cumbersome, they would be unlikely to participate. Some stated that the whole point of LTAs is to be convenient, and as such, ones that require extensive setup or input defeat their own purpose. Others said that knowing themselves, they would not adhere to a difficult or laborious task for very long, if at all. Interestingly, however, participants did mention they would be willing to tolerate a certain level of inconvenience in the context of research, particularly if the topic was interesting to them. This may be since, often when participating in research experiments, participants are being given something in return (financial compensation or course credits) that increases their tolerance. Researchers would benefit from (a) designing or utilising LTAs that cause little inconvenience, such as “passive” LTAs, and (b) offering a higher compensation to persuade otherwise reluctant students, particularly if the study has a long duration.

When it came to intrusiveness, annoyance was again a commonly mentioned emotion. Participants disliked the idea of being “pinged” or receiving many notifications, with some saying they would begin to view the experiment as a “chore.” This was explored further in the hypothetical scenario, asking participants how often they would answer mental health questionnaires *via* the LTA before it would be considered too intrusive. Participants typically said that anything more frequent than *once a week* would be considered too intrusive to their daily lives, which is likely too limited a rate to be feasible for data collection. Similarly, to inconvenience, however, participants may be willing to tolerate intrusiveness if financially compensated. Indeed, when asked if they would participate in a “maximal” version of the hypothetical scenario (where they answered questionnaires several times a day), all participants were willing should they receive money for doing so.

Building off this, participants almost all prioritised financial compensation over additional course credits as hypothetical incentives, suggesting that monetary rewards may be an extremely powerful way of increasing intake. University students on average have less income than other demographics ($24,249 compared to the median salary of ∼$51,000 in 2019; NZSTAT, 2019), meaning they may be more motivated by the chance to supplement it. This may be intensified by the abnormally high increases for cost-of-living seen by recent geopolitical events (6.9% increase in total living costs between March 2022 quarter and December 2021 quarter). As such, researchers will likely see increased intake from any financial compensation they can incorporate.

Finally, researchers may benefit from “playing to their strengths” regarding whether they are from the academic or corporate sector. Some students favoured the idea of a small university-based team, citing the personal level that their contribution would impact (e.g., helping a researcher complete their thesis) and the focus on research for the sake of research, rather than exploitation. Other students were more likely to participate if a large company was conducting the experiment, as their increased resources would make them more capable of handling privacy and data security concerns, as well as the prestige of being involved in a “big name” company. Researchers may wish to tailor their advertisements to evoke these traits (e.g., emphasising how participation will help the researchers or name dropping a famous LTA/company) and thus attract participants.

As mentioned previously, research into the acceptability of passive mobile sensing and LTAs is relatively scant but reveals some shared insights into those found in this study as well as several unique contributions of this study. As seen both in this study and previous studies, privacy concerns are highly important for one's acceptance and comfort with mobile sensing data collection (12). The trend for privacy to increase in perceived importance for more sensitive data compared to activity or sleep data seen in this study has also been observed previously (19). Previous findings also suggested young people are reasonably comfortable sharing their data (15), which may be supported by the ambivalence some participants expressed in this study, although this was not a universal opinion. The general cynicism regarding actually trusting researchers regarding data privacy and security has been observed before (20) and is not surprising given the multitude of infamous scandals regarding breaches of trust. It is paramount that researchers take every effort to foster a sense of trust in participants if they wish to maximise adherence and participation. Finally, this study supported previous findings that inconvenience and hassle (e.g., battery drainage) are important influences on perceived feasibility and thus usage by participants (12). However, this study extracted these findings in the unique context of university students considering whether to participate in LTA *research*, as opposed to other demographics engaging with commercial products for their everyday life. As such, these results have high ecological validity for researchers aiming to increase participant uptake for their studies and can better inform decision making regarding recruitment and retainment.

This study had limitations. Given LTAs themselves are a nascent development in technology, this paper examined the manifest content of the data with little examination of the underlying, as to be conservative with insight generation. Future studies may wish to instead examine the latent content (a higher level of interpretation and abstraction) to uncover greater detail, using this study and others as grounds for potential to examine.

This study did face the limitation of a small sample size: only 21 participants signed up to the study and were able to make the agreed upon timeslots. Additionally, the sample has a significant gender imbalance (1:7 male to female ratio) and was collected only from Otago University. As such, insights from this study may not be easily applied to other demographics. For example, with a greater sample of male participants may come a gendered effect of whether privacy matters to participants. Other cultures may also show different stances regarding privacy, data security, or inconvenience: collectivistic cultures often place a priority on group needs (such as universities needing research to be completed) and as such may not view personal inconvenience as an issue needing compensation. Studies examining the same topic, both again for New Zealand university students and for other student demographics, would be beneficial for replicability and generalisability. Finally, the researchers directly asked participants about some topics (e.g., privacy, data security, inconvenience, intrusiveness). This may have led participants to overly talk about these factors in relation to their decision making, even if there were others that are important. This was mitigated somewhat by asking participants open-ended questions at the end of the experiment (e.g., “is there anything else that would make you more or less likely to participate”) but may remain an issue. Future studies may wish to use a more unstructured design to their questionnaires, but this approach has its own risks of receiving off-topic replies.

## Conclusion

This study aimed to conduct a qualitative investigation into what may make university students more or less likely to participate in research using LTAs installed on their smartphones. Participants offered vital insights regarding expected categories of privacy, data security, inconvenience, and intrusiveness, while also generating novel categories such as financial compensation and academic vs. corporate researchers. These insights will hopefully be utilised by researchers to maximise intake for LTA-based experiments, and in turn help push the field forward to maximise acceptability and uptake.

## Data Availability

The raw data supporting the conclusions of this article will be made available by the authors, without undue reservation.
