# Supplementary material for: University students’ opinions towards mobile sensing data collection: A qualitative analysis
Source: Front Digit Health. 2023 Apr 13;5:1125276. doi: 10.3389/fdgth.2023.1125276 (PMC10134000; doi:10.3389/fdgth.2023.1125276)
Supplement: Supplementary file 1 [file Datasheet1.pdf]

COREQ 32 ITEM CHECKLIST

No	Item	Guide questions/descriptions	Response
**Domain 1: Research team and reflexivity**			
Personal Characteristics			
1.	Interviewer/facilitator	Which author/s conducted the interview or focus group?	Jack R.H. Cooper
2.	Credentials	What were the researcher's credentials? *E.g. PhD, MD*	Bachelor of Psychology (Hons)
3.	Occupation	What was their occupation at the time of the study?	Completing PhD (student)
4.	Gender	Was the researcher male or female?	Male
5.	Experience and training	What experience or training did the researcher have?	No experience/training conducting focus groups
Relationship with participants			
6.	Relationship established	Was a relationship established prior to study commencement?	No
7.	Participant knowledge of the interviewer	What did the participants know about the researcher? e*.g. personal goals, reasons for doing the research*	Occupation, credentials, reasons for conducting the research (understanding the opinions and experiences participants had had with LTAs, and what may make them more likely to participate in LTA research
8.	Interviewer characteristics	What characteristics were reported about the	Reasons and interests in the research topic (potentially relevant to their own research)

		interviewer/facilitator? e.g. *Bias, assumptions, reasons and interests in the research topic*	
**Domain 2: study design**			
Theoretical framework			
9.	Methodological orientation and Theory	What methodological orientation was stated to underpin the study? *e.g. grounded theory, discourse analysis, ethnography, phenomenology, content analysis*	Qualitative content analysis (inductive/phenomelogical/manifest content approach)
Participant selection			
10.	Sampling	How were participants selected? *e.g. purposive, convenience, consecutive, snowball*	Purposive (research question was specifically regarding university students)
11.	Method of approach	How were participants approached? e.*g. face-to-face, telephone, mail, email*	Online website advertisement (StudentJobSearch.com)
12.	Sample size	How many participants were in the study?	21
13.	Non-participation	How many people refused to participate or dropped out? Reasons?	None: 11 did not show up to session despite signing up
Setting			

14.	Setting of data collection	Where was the data collected? e.*g. home, clinic, workplace*	1 session in person (Daily Experiences Lab, Leith 270, University of Otago), 4 online over Zoom (due to the then active COVID outbreak in New Zealand)
15.	Presence of non-participants	Was anyone else present besides the participants and researchers?	No
16.	Description of sample	What are the important characteristics of the sample? *e.g. demographic data, date*	Demographics (university students, uneven gender ratio)
Data collection			
17.	Interview guide	Were questions, prompts, guides provided by the authors? Was it pilot tested?	Questions/prompts/guides were provided by the authors both orally and written (paper copies/online copies). They were not pilot tested, but were approved by Tamlin Conner (co-author and supervisor to Jack R.H. Cooper)
18.	Repeat interviews	Were repeat interviews carried out? If yes, how many?	No
19.	Audio/visual recording	Did the research use audio or visual recording to collect the data?	Yes: Session 1 collected audio data and Sessions 2-5 collected audio+visual data via Zoom recording feature.
20.	Field notes	Were field notes made during and/or after the interview or focus group?	Yes
21.	Duration	What was the duration of the interviews or focus group?	~45-60 mins
22.	Data saturation	Was data saturation discussed?	Yes
23.	Transcripts returned	Were transcripts returned to	No

		participants for comment and/or correction?	
**Domain 3: analysis and findings**z			
Data analysis			
24.	Number of data coders	How many data coders coded the data?	1
25.	Description of the coding tree	Did authors provide a description of the coding tree?	Yes
26.	Derivation of themes	Were themes identified in advance or derived from the data?	Derived from data, except for Category 3 and it's subthemes (privacy, data security, intrusiveness and inconvenience) which were conceptualised in advance
27.	Software	What software, if applicable, was used to manage the data?	Otter.ai (transcribed audio data)
28.	Participant checking	Did participants provide feedback on the findings?	No
Reporting			
29.	Quotations presented	Were participant quotations presented to illustrate the themes / findings? Was each quotation identified? e.*g. participant number*	Yes
30.	Data and findings consistent	Was there consistency between the data presented and the findings?	Yes
31.	Clarity of major themes	Were major themes clearly presented in the findings?	Yes
32.	Clarity of minor themes	Is there a description of diverse cases or discussion of minor themes?	Yes
